# Downward comparisons enhance carbon credit policy sustainability: Lab evidence

**DOI:** 10.1016/j.isci.2025.114050

**Published:** 2025-11-13

**Authors:** Chenke Hu, Kehua Wang, Linghao Wang, Jun Zhao, Zheng Zhu, Hai Yang, Hongxing Ding, Guibing He, Ziyi Wang

**Affiliations:** 1 Institute of Intelligent Transportation Systems & Polytechnic Institute, Zhejiang University, Hangzhou, China; 2 Institute of Intelligent Transportation Systems, College of Civil Engineering and Architecture, Zhejiang University, Hangzhou, China; 3 Department of Civil & Architectural Engineering and Mechanics, University of Arizona, Tucson, AZ, USA; 4 Department of Civil and Environment Engineering, Hong Kong University of Science and Technology, Hong Kong, China; 5 Department of Psychology and Behavioral Sciences, Zhejiang University, Hangzhou, China; 6 Zhejiang Tranalytic Technology Company, Wenzhou, China

**Keywords:** Environmental science, Environmental management, Environmental policy

## Abstract

Carbon-inclusive policies that modify daily activities are crucial for reducing emissions. The Credit Charge-cum-Rebate (CCR) scheme is a promising approach to encouraging pro-environmental behavior, balancing fiscal and emission performance. However, it remains theoretically underexplored. To address this, an experimental study with 270 participants was conducted. A validation experiment identifies significant theory-practice deviations, particularly in the low-credit group, which jeopardizes fiscal sustainability. Three non-financial interventions are tested, with low-credit feedback using downward social comparison proving most effective at improving fiscal outcomes. A Beijing-based case study illustrates potential savings of billions of yuan over six years. The study recommends that policymakers implement targeted interventions such as downward comparisons for penalized low-credit users, and integrate non-financial tools more widely.

## Highlights

• Commuting decisions under carbon credit schemes (CCR) deviate from full rationality
• Downward social comparison feedback effectively enhances the behavioral stability of CCR
• Beijing case study projects potential savings of billions with targeted interventions
• Tailored interventions for distinct groups are advocated, with attention to ethical risks

## Graphical abstract

**Figure:**
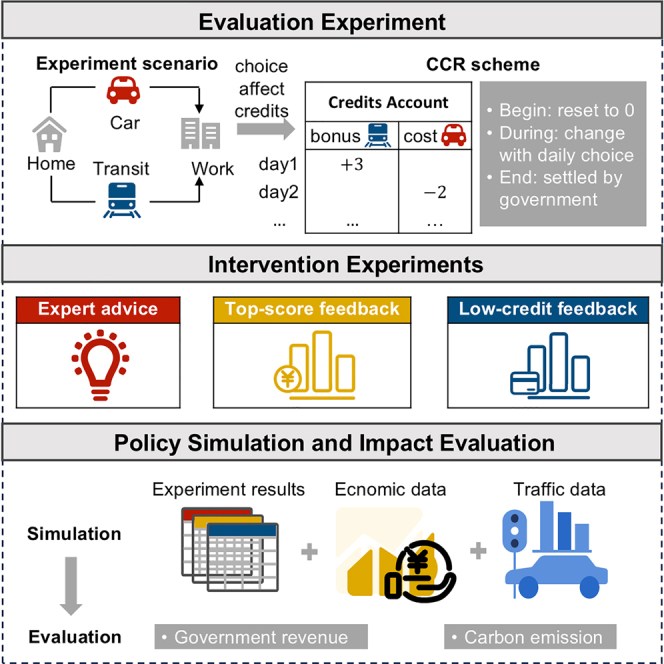

## Introduction

### Contexts and literature review

The Paris Agreement’s overarching objective is to achieve global net-zero carbon dioxide emissions by 2050. Among the various strategies for carbon mitigation, policies aimed at influencing human daily life behavior are the most sustainable and effective.^1^

Administration means, such as vehicle restriction policies, are a widely adopted approach to influence daily behaviors. Owing to their coercive nature, such measures often produce only short-term outcomes and may induce behavioral adaptation, including unintended consequences such as the acquisition of additional vehicles.

Fiscal instruments are another effective category of policies. Mainstream fiscal tools include congestion pricing, the carbon tax, the Cap-and-Trade emissions trading system, and subsidy-based incentive mechanisms. Carbon taxation and congestion pricing are based on the theory of negative externalities—Pigovian taxation,^2^ while the subsidy mechanism is grounded in the theory of positive externalities. Now, carbon tax and subsidy methods have been widely implemented in many countries. However, these programs often encounter challenges related to equity issues, public acceptance, budgetary constraints, and uncertain effectiveness.^3^ For example, Singapore’s electronic road pricing (ERP) system is a market-based fiscal policy that regulates traffic congestion by charging vehicles for using congested roads during peak hours.^4^ However, while it addresses congestion efficiently, it has been criticized for disproportionately impacting low-income drivers who cannot afford the tolls. Therefore, an increasing number of researchers have turned their attention to the Cap-and-Trade scheme. The Cap-and-Trade scheme is grounded in Coase Theory^5^ and the theories of market-based externality regulation. Its central principle involves establishing an emissions ceiling and allowing the market to allocate emissions allowances through trading.^6^ Currently, Cap-and-Trade frameworks primarily operate at the corporate and institutional levels.

In transportation, policy researchers have also introduced behavioral regulation measures that monetize carbon emissions, such as the Tradable Credit Scheme (TCS), to control the number of vehicular travelers. The TCS adopts the Cap-and-Trade principle to regulate individual transportation-related emissions.^7^ With such a scheme, the government allocates certain credits periodically for trips to eligible travelers. Credits must be surrendered when travelers use private cars, and they are free to trade credits in the market. This design maintains government revenue neutrality and alleviates public concerns about fairness. TCS has attracted considerable academic attention since its proposal, with studies expanding mainly on its policy design, market efficiency, and environmental effectiveness.^8^^,^^9^

The Credit-Charge-cum-Reward (CCR) scheme, which is the focus of this study, is a TCS variant.^10^ The key difference between CCR and TCS is the prohibition of peer-to-peer credit trading; residents are only allowed to settle carbon credits with the government at the end of each cycle, and credit prices are fixed. Furthermore, it combines rewards and penalties, incentivizing low-carbon behaviors while penalizing high-carbon behaviors. This is similar to the core principle of China’s ECS system.^11^ Indeed, the CCR is essentially a specific implementation of China’s environmental credit scoring system in the transportation sector.

The TCS faces complex challenges in policy design and challenges in ensuring equitable allocation.^12^ In contrast, the CCR avoids market mechanism volatility and uncertainties, resulting in high institutional controllability and feasibility.^13^ It retains the behavioral incentive structure of China’s ECS while introducing a clear fiscal framework. Unlike the Singapore ERP, it avoids direct congestion charges that disproportionately affect specific groups. Furthermore, the CCR offers greater flexibility in fiscal management and carbon emission control due to the combined reward-and-penalty structure. By adjusting its key parameters, policymakers can achieve a balance between fiscal objectives and carbon targets.^10^ Thus, the CCR scheme demonstrates strong fiscal neutrality and institutional controllability advantages, making it a highly promising carbon-inclusive policy. Although initially proposed in the transportation sector, it has broad applicability extended to other fields, such as daily energy consumption.^14^

However, current research on the CCR is still at the stage of theoretical modeling, with no empirical validation. To address this gap and investigate individuals' behavioral characteristics under the CCR scheme, several experiments, including validation and intervention experiments, are conducted using a self-developed experimental platform. Behavioral experiments offer the advantages of low cost, rapid implementation, and better control of scenarios, making them suitable for evaluating the effectiveness of new policies. In recent years, many scholars have used experiments to study transportation issues such as route choice, departure time selection, and mode choice.^15^ The heterogeneity in travelers' value of time (VOT), which denotes the monetary value of time spent traveling and is a key factor reflecting people’s sensitivity toward CCR,^16^ has been taken into account in the experiments of this study.

### Validation experiment

**Figure 1:**
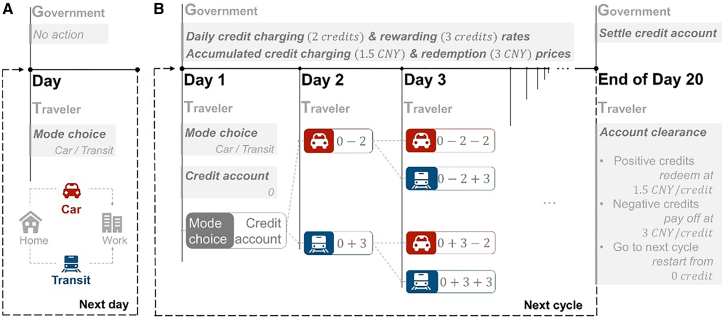
The operational framework of the experiments (A) The experimental process without the CCR. (B) The experimental process with the CCR, with parameters in the experiments.

Participants are required to make travel mode choices in a simulated commuting scenario in the validation experiments. The scenario has one transit route (with high travel time but low travel fees) and one highway route (with low travel time but high travel fees) connecting residential areas to workplaces (Figure 1A). On each day, the highway travel time of a car trip is monotonically increasing with the flow of highway traffic (i.e., the number of car users), and the detailed formula is given in Section Theoretical equilibrium. The fee for each car trip is 14 CNY (Chinese Yuan). The travel time and fee for each transit trip are 60 min and 4 CNY, respectively. Under the CCR, participants are informed of the implementation of the scheme (see Figure 1B): The scheme is implemented on a periodic basis (the cycle) for 20 days. At the beginning of the cycle, all travelers have access to the CCR scheme by simply having a credit account with zero balance. During the cycle, travelers will be charged 2 credits for each car trip and rewarded 3 credits for each public transit trip. Each traveler aims to minimize the individual periodic travel cost for the 20 days by adjusting his/her daily travel mode and the daily credit consumption or acquisition. At the end of the cycle, the government settles and clears the accumulated credits in each traveler’s account. Participants with a credit surplus will obtain incentives valued at the redemption price of 1.5 CNY/credit, whereas those with a deficit will pay off the deficit at the charging price of 3 CNY/credit. Each experiment involves 15 participants, simulating 6 cycles, corresponding to 120 days of choices. Participants are assigned their own VOT value (20 CNY/h, 36 CNY/h, 50 CNY/h), which divides them into three VOT groups (the low-, medium-, and high-VOT groups). Participants' rewards are negatively correlated with their travel costs (modeled as a linear sum of fees, time costs, and costs from the credit settlements; specific formulas are detailed in Section Theoretical equilibrium), guiding them toward making rational choices.

**Figure 2:**
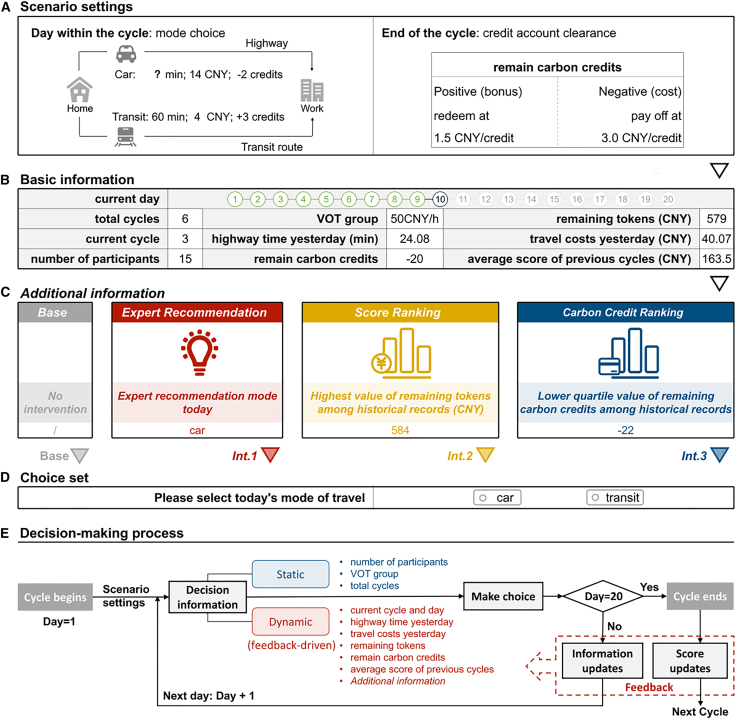
Information on the experimental platform interface and the decision-making process of participants (A) Scenario Information. (B) Basic feedback information in four experiments. (C) Additional feedback information in four experiments. (D) Selection box. (E) Decision-making process of participants.

In the validation experiment (labeled *Base* later, and Figures 2A, 2B, and 2D show its experimental interface), participants made choices each day based on the feedback information provided on the screen. Static feedback information involves scenario settings and parameters of the CCR, total cycles, VOT, and number of participants. Dynamic feedback information includes the current cycle and day, the highway travel time for the previous day, travel costs for the previous day, the remaining tokens (calculated by subtracting travel costs from the initial tokens), remaining carbon credits, and the average score of previous cycles. The decision-making process of the subjects within a cycle is shown in Figure 2E. Information is provided as feedback at the end of each day and cycle; therefore, the subjects learn both within and between cycles.

The validation experiments aim to explore the gap between theory and practice. The following hypotheses are preregistered:

Research Question 1: Do the travel mode choices of individuals under the CCR align with theoretical predictions (i.e., are individuals fully rational)?

Hypothesis 1 (H1): Under the CCR, individuals are rational. The driving proportion in the experiment differed

from that in the theoretical predictions.

Clarifying how fully rational and rational behaviors are defined in this study is necessary. Rational behavior refers to travel mode choice decisions that aim to minimize an individual’s total travel costs. The assumption of fully rational behavior forms the core of classical traffic equilibrium theory—user equilibrium—which is widely applied to predict and explain traffic flow patterns.^17^ The theoretical model of the CCR relies on this assumption, under which individual mode choices are expected to converge toward a user equilibrium, a state where no traveler can unilaterally change modes and further reduce costs. However, in reality, individuals often face cognitive constraints, limited information processing capacities, emotional influences, or social norms, leading them to adopt satisfying strategies, i.e., they select solutions that are “adequate” rather than optimal. Such deviations from the user equilibrium are typically referred to as bounded rationality.^18^ In this study, it is hypothesized that the feedback of carbon credits may influence rational decision-making.

The validation experiments confirmed significant deviations between theoretical predictions and actual car-use behaviors of participants, especially in the high-VOT groups. Although these deviations enhance carbon reduction performance, they pose fiscal risks for governments that base budget planning on theoretical predictions, potentially undermining the sustainability of the scheme. To address this challenge, three non-financial interventions are designed, and the corresponding experiments are conducted. Non-financial measures are distinguished by their low cost and high public acceptability, making them a practical tool for behavioral change.^19^^,^^20^ However, their impact is often viewed as constrained, suggesting that they complement financial mechanisms.^21^

### The intervention experiment

In contrast to the validation experiments, three types of additional feedback information are shown to participants (see Figure 2C) in the intervention experiments: (1) The Expert Recommendation experiment (labeled *Int.1* later) recommends theoretical optimal mode; (2) The Top-Score Feedback experiment (labeled *Int.2* later) displays the highest remaining tokens in the records; (3) The Low-Credit Feedback experiment (labeled *Int.3* later) shows the third quartile of carbon credits in the records for the high-VOT participants. These three interventions are designed to guide participants in making rational choices. Notably, *Int. 3* aims to nudge downward comparisons and instill the perception among high-VOT individuals that “my carbon credits are higher than some peers”, thereby alleviating the psychological burden associated with reduced carbon credits when choosing to drive. The following hypotheses are preregistered:

Research Question 2: Can the intervention measures alter travel mode choices?

Hypothesis 2.1 (H2.1): Under the CCR policy, all three intervention measures significantly influence travel mode choices. The driving proportions of the system under each intervention condition are higher than those in the baseline condition.

Hypothesis 2.2 (H2.2): *Int.3* produces the most pronounced effect on the choice of travel mode. The driving proportion of the system reaches its highest level under *Int. 3*.

The rationale for these interventions' effectiveness primarily draws on the broad advantages and empirical support of “nudge” strategies in behavioral economics. A substantial body of research has shown that nudges—non-coercive, low-cost interventions—can effectively guide individual behaviors by altering choice architecture while preserving individual autonomy.^22^

Nevertheless, any behavioral intervention carries potential negative outcomes that must be considered. For instance, while expert recommendations have authority, if perceived as nontransparent or coercive, they may trigger resistance or reduce trust.^23^ Downward comparisons, through alleviating some negative emotions, can lead to complacency and behavioral slack if they are misapplied.^24^ Frequent feedback may increase cognitive burden and reduce decision efficiency.^25^ Despite these concerns, the theoretical foundations and existing empirical evidence provide ample reason to expect that these interventions significantly influence travel mode choices.

Given that *Int. 3* specifically addresses the psychological mechanisms of high-VOT travelers, its effects are expected to be particularly pronounced.

Exploratory Question: How do different intervention strategies affect learning behaviors?

Understanding how individuals adjust their travel behaviors through learning is crucial for evaluating the policy’s long-term effectiveness in the complex context of the CCR. Learning curve of driving proportions and the learning curve of switching proportions over days and cycles are used to analyze individuals' learning behaviors. Observing learning behaviors under different strategies helps reveal the mechanisms through which these strategies take effect.

The results from the intervention experiments indicate only the stable driving ratio under *Int. 3* is significantly higher than that under *Base*. However, all three interventions shift the government’s fiscal position from a deficit to a surplus. Among these, *Int.3* generates the highest fiscal revenue for an equivalent increase in carbon emissions.

### Predictive performance of interventions in Beijing

Finally, a case study focusing on the central urban area of Beijing during the morning peak is conducted to evaluate the interventions' urban-scale benefits. The results show that over six years, the three interventions can save costs amounting to 0.74 billion CNY, 1.42 billion CNY, and 2.4 billion CNY, respectively.

## Results

**Figure 3:**
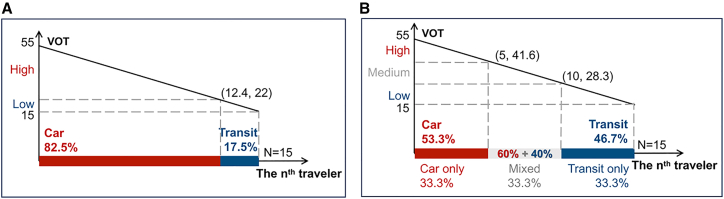
Theoretical derivation results of the CCR scheme (A) Theoretical equilibrium without the CCR scheme. (B) Theoretical equilibrium with the CCR scheme.

The theoretical benchmark is outlined before the analysis. Without CCR, the predicted CO_2_ emission is 77.1 g per capital per cycle. Under CCR, the predicted driving proportions of the three groups are 0, 0.6, and 1, respectively. The predicted carbon emission decreases to 31.5 g per capital per cycle, and fiscal revenue is projected at 10 CNY per capital per cycle. (See Section theoretical equilibrium and Figure 3 for details on theoretical equilibrium).

### Convergence of the system (questions 1 and 2)

The three main metrics are the stable driving proportion, stable carbon emissions, and stable financial balances of the system. The driving proportion is calculated as the ratio of the number of car trips to the total number of travel trips. The stable value is the average value of the last two cycles (which is a relatively stable stage).

**Figure 4:**
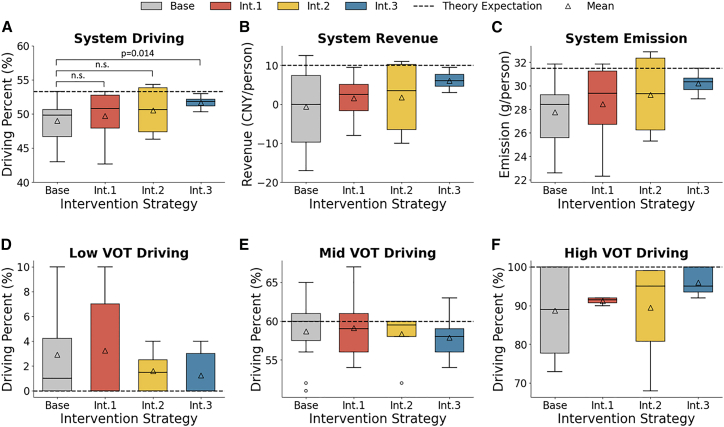
Static results of experiments *t* test hypothesis testing is utilized to evaluate the stable metrics. The significance levels are represented as “ns” for *p* > 0.05. In the boxplot, the center line represents the median, the box spans the interquartile range (IQR) from the 25th to the 75th percentile, and the whiskers extend to the minimum and maximum values within the dataset. (A) (D)–(F)The stable value of the driving frequency for four strategies in the system and three groups. (B) Fiscal revenue per cycle under four strategies. (C) CO2 emissions per cycle under four strategies.

First, the whole system’s stable driving proportion is examined (Figure 4A) to verify the discrepancies between the experimental results and theoretical predictions. The t-hypothesis testing indicates that the stable driving proportion in *Base* is significantly lower than the theoretical value of 0.53 (*M*_Base_ = 0.49, SD = 0.031, 95% CI, [0.470, 0.510]; *p* = 0.001]). This finding indicates that travelers are not entirely rational; more precisely, their decisions reflect bounded rationality. (The *H1* is supported).

Second, the differences between the various groups in *Base are* analyzed. Figures 4D–4F illustrates the stable driving proportions of different groups. Experimental results reveal deviations from theoretical predictions of less than 3% in the low VOT group ($M_{\text{Base}\_\text{low}}=0.029$, SD = 0.095, 95% CI, [0, 0.065]) and the medium VOT group ($M_{\text{Base}\_\text{med}}=0.587$, SD = 0.090, 95% CI, [0.553, 0.620]). However, the high VOT group ($M_{\text{Base}\_\text{high}}=0.884$, SD = 0.187, 95% CI, [0.812, 0.955]) deviates from the theoretical value by more than 10%. These findings suggest that the gap between actual and theoretical outcomes under the CCR scheme primarily stems from the high VOT group.

Next, system’s carbon emissions and fiscal revenue are focused (see Figures 4B and 4C). Compared with the theoretical prediction of 77.1 g without CCR, the actual carbon emissions are reduced by 64% (M_Base_ = 27.8, SD = 2.85, 95% CI, [26.0, 29.6]). Compared with the predicted emission under CCR, the carbon emissions are 11.8% lower in *Base*. However, the experimental financial performance deviates entirely from the predicted results, transitioning from a predicted revenue of 10 CNY per person per cycle to an expenditure of 0.583 CNY (SD = 0.471, 95% CI, [-4.819, 3.652]), a decline of 105%.

First, the stable driving proportions of various interventions are examined. When comparing the systematic stable driving proportions (see Figure 4A), the driving proportions under the three interventions all increase from *Base* (M_I1_ = 0.497;M_I2_ = 0.505;M_I3_ = 0.517). However, the t-hypothesis testing demonstrates that only *Int.3* has a significantly higher driving proportion than *Base*, while *Int.1* and *Int.2* show no significant differences from *Base*, while *Int.3* has a significantly higher driving proportion than *Base* ($p_{I1-\text{base}}=0.655;$ $p_{I2-\text{base}}=0.322;$ $p_{I3-\text{base}}=0.014$). Here, *H2.1* is partially supported, while *H2.2* is supported. An analysis of the stable driving proportions by group reveals that participants with high and low VOT are most strongly influenced by *Int.3*, while those with medium VOT are primarily associated with *Int.1.* (see Figures 4E and 4F).

From the perspective of the system’s emission reductions and revenue (see Figures 4B and 4C), all interventions shift the system’s financial situation from expenditure to revenue generation. The revenue generated by *Int.3* (*M*_*I*3_ = 6.06, SD = 2.24, 95% CI, [4.19, 7.94]) is nearly four times that of *Int.1* (*M*_*I*1_ = 1.56, SD = 5.56, 95% CI, [-3.08, 6.21]) and *Int.2* (*M*_*I*2_ = 1.75, SD = 9.61, 95% CI, [-6.29, 9.79]). Calculated as additional carbon emissions per unit of additional revenue, the cost-effectiveness ratios of the three interventions are 0.33, 0.68, and 0.259 g per CNY, respectively, showing that *Int.3* performs best in improving CCR’s fiscal sustainability.

### Dynamic selection process (exploratory question)

Dynamic choosing-process analysis in this study is crucial for understanding how participants adapt their choices over time. The calculation method of driving proportions has already been explained in the Introduction section. If an individual’s mode choice differs from the previous day, he/she is considered a switching mode commuter. Switching proportions are calculated as the ratio of commuters switching to the total number of commuters.

**Figure 5:**
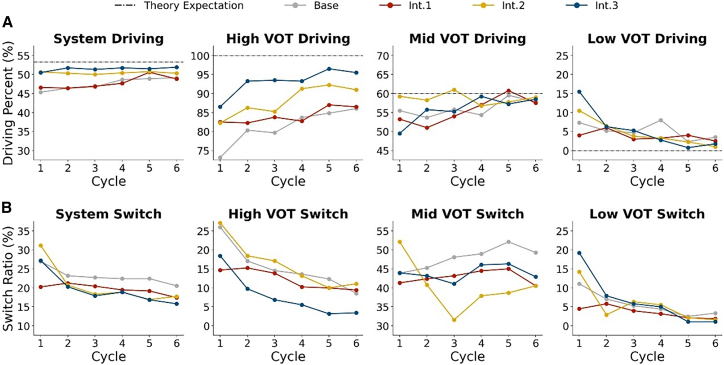
Dynamic results of experiments (A) Cycle-by-cycle variation curve of driving proportions. (B) Cycle-by-cycle variation curve of the average switching ratio.

The variation in driving proportions across six cycles is examined to evaluate the learning speeds of participants. (Figure 5A).

First, the driving proportion curves in *Base* are analyzed. The driving proportions fluctuated over time, but it can reach a stationary state after about 4 cycles in both the system and groups. The medium-VOT group demonstrates the fastest convergence to the theoretical prediction, approaching the theoretical equilibrium value by cycle 5. In contrast, the low-VOT group remains 3% away from its predicted value by the fifth cycle, while the high-VOT group significantly lagged, maintaining a 16% deviation from its theoretical target. These results suggest that the low-VOT and high-VOT groups exhibit slower learning speeds than the medium-VOT group. In the high-VOT groups, the driving proportion in the first cycle is significantly lower than predicted, indicating strong bounded rationality in their choices. A sharp increase in driving is observed in the second cycle, suggesting that the end-of-cycle score feedback has a substantial impact on their decisions. As the participants continue to receive score feedback in subsequent cycles, their choices gradually become more rational.

*Int.2* and *Int*.3. However, in the last three cycles, the driving proportions under *Int. 2 showes* no significant difference from those of *Base* (Mann-Whitney *U* test, *p-*value >0.1), whereas *Int.* 3 maintains a significant difference (Mann-Whitney *U* test, *p*-*value* < 0.1). This finding highlights that *Int.2*’s strong short-term effectiveness of Int.2 but its limited long-term impact.

Then, the results from a group-specific perspective are analyzed to compare the impact of different interventions using the Mann-Whitney U test. For the high-VOT group, the results indicate that the driving proportions under *Int.2* and *Int.3* of six cycles are all significantly higher than *Base* (Mann-Whitney *U* test, *p*-value <0.05), suggesting that both *Int.2* and *Int.3* not only improve the convergence outcomes for high-VOT participants but also accelerate their learning speed. For the medium-VOT group, the results indicate that during the first three cycles, only the driving proportions under *Int.2* significantly outperform *Base* (Mann-Whitney U test, *p-*value <0.1), highlighting *Int.2’s* strong short-term influence on medium-VOT participants. However, the *Int.2* curve experiences a steep decline in the third cycle, eroding its early advantage. This trend aligns with the system-level driving proportion curve under *Int.2*, indicating that the instability of *Int.2* primarily originates from the medium-VOT group.

The system stability is assessed by analyzing the mode-switching frequencies over six cycles (Figure 5B). A lower switching proportion generally indicates higher internal stability.

First, in *Base*, determining whether the switching proportion of the system stabilizes after six cycles is difficult. Group-level analysis reveals that the switching frequencies of both the high-VOT and low-VOT groups stabilize below 0.1 after six cycles. In contrast, the medium-VOT group maintains a substantially higher switching frequency of 0.48, suggesting greater behavioral instability. This is likely driven by stronger influence from day-to-day competition with others. A sharp drop in switching proportion is observed in the second cycle among high-VOT participants, resulting from their sharply increased tendency to choose driving in that cycle.

Then, the switch proportions between the interventions are compared. The results of the Mann-Whitney *U* tests show that all three interventions yield significantly lower switching proportions of the system compared to *Base* (Mann-Whitney *U* test, *p-value* < 0.1), demonstrating improved system stability. For the high-VOT group, only the switching proportions under *Int.3* are significantly lower than those of *Base* within six cycles (Mann-Whitney *U* test, *p-value* < 0.1), indicating that the high-VOT system under *Int.3* achieves the highest stability. For the medium-VOT group, *Int.2* initially causes a sharp increase of 10% in switching frequency compared to *Base* during the first cycle, reflecting heightened instability in the early stage.

### Decision modeling (Questions 1 and 2)

As shown in Section convergence of the system (questions 1 and 2), the deviation from theoretical predictions is the greatest among high-VOT individuals. It is hypothesized that the absolute value of carbon credits may influence decisions due to psychological factors such as environmental awareness or aversion to negative credits. Logit models are established to explore the assumption. The cost variables and the credit variables are selected as explanatory variables. The explained variable is the mode of choice, coded as 1 if driving is chosen and 0 otherwise.

The cost variables include daily driving and transit costs. Cost calculations incorporate monetary expenses, time costs, and costs arising from carbon credits changes. The cost due to changes in carbon credits is derived by subtracting the settlement cost before and after the choice.

According to the scenario settings in Section validation experiment, the range of carbon credits is [-40, 60]. These carbon credits are divided into 20 intervals at 5-point increments, with each interval treated as a separate variable. After filtering out variables with col-linearity >0.8, constant-valued variables, and variables that fail the correlation test, three variables, *C*_[-5,0)_, *C*_[-20,-15)_, and *C*_[0,5)_, remain. They are defined to indicate whether the pre-choice carbon credits fall within the ranges [-5, 0), [-20, −15), and [0, 5), respectively; and the three credit levels indicate the states of mild deficit, mild surplus, and significant deficit, correspondingly. The logit model results are as follows (as shown in Table 1):

**Table 1:** Summary of logit models across the four strategies

Base	Sample = 3600, *R*^2^ = 0.263				
	Coef.	Std. Err.	[0.025	0.975]	*p*-value
Const.	−20.03	2.45	−24.84	−15.22	0.00
Car Cost	0.60	0.04	0.53	0.68	0.76
Transit Cost	−0.06	0.03	−0.12	0.01	0.97
*C*_[-5,0)_	−0.60	0.13	−0.85	−0.36	0.00
*C*_[-20,-15)_	0.86	0.18	0.50	1.22	0.00
*C*_[0,5)_	2.43	0.25	1.95	2.91	0.00

**Int.1**	Sample = 2400, R^2^ = 0.271				
	Coef.	Std. Err.	[0.025	0.975]	*p*-value
Const.	−14.88	3.18	−21.11	−8.66	0.00
Car Cost	0.53	0.05	0.43	0.62	0.75
Transit Cost	−0.10	0.04	−0.18	−0.02	0.65
*C*_[-5,0)_	−0.59	0.16	−0.90	−0.28	0.00
*C*_[-20,-15)_	0.58	0.22	0.16	1.01	0.01
*C*_[0,5)_	3.32	0.43	2.48	4.16	0.00

**Int.2**	Sample = 2400, R^2^ = 0.176				
	Coef.	Std. Err.	[0.025	0.975]	*p*-value
Const.	−12.82	3.24	−19.17	−6.46	0.00
Car Cost	0.50	0.05	0.40	0.61	0.90
Transit Cost	−0.12	0.05	−0.21	−0.04	0.77
*C*_[-5,0)_	−0.51	0.17	−0.84	−0.19	0.00
*C*_[-20,-15)_	1.00	0.26	0.50	1.50	0.00
*C*_[0,5)_	1.52	0.23	1.07	1.97	0.00

**Int.3**	Sample = 2400, R^2^ = 0.234				
	Coef.	Std. Err.	[0.025	0.975]	*p*-value
Const.	−20.77	4.04	−28.68	−12.86	0.00
Car Cost	0.50	0.06	0.37	0.62	0.85
Transit Cost	0.06	0.06	−0.06	0.18	0.35
*C*_[-5,0)_	−0.36	0.20	−0.75	0.02	0.07
*C*_[-20,-15)_	−0.27	0.38	−1.03	0.48	0.48
*C*_[0,5)_	3.92	0.59	2.76	5.08	0.00

Std. Err.: The standard error of the coefficient estimate. [0.025 | 0.975]: Lower and upper bounds of the 95% confidence interval for the coefficient.

In the base experiment, cost does not exhibit a significant association with choices, whereas current carbon credit levels do. When carbon credits are within [-5, 0), the probability of choosing driving decreases. On the contrary, when within [0, 5), the probability increases. Once credits lie within [-20, −15), the probability of driving increases, indicating that individuals with a history of high driving frequency are more likely to continue driving.

The coefficient magnitude of *C*_[-5,0)_ can be used to compare the extent to which the carbon neutrality mentality influences participants' choices under different strategies. After implementing interventions, the magnitude of *C*_[-5,0)_decreases, implying a reduced inhibitory impact of carbon credits on driving. Moreover, the coefficients of *C*_[-20,-15)_ under all other strategies are positive and statistically significant, but under Int.3, the coefficient is not statistically significant.

### Policy simulation in Beijing

**Figure 6:**
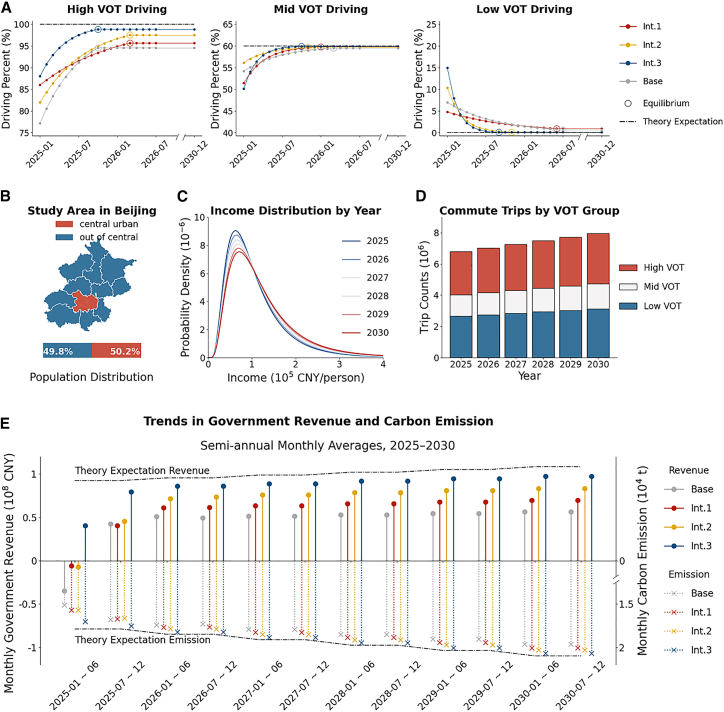
Forecast outcomes of the Beijing case study (A) Evolution curve of the driving proportion from 2025 to 2030. (B) Geographic scope and population proportion of Beijing’s central urban area. (C) The income distribution curve from 2025 to 2030. (D) Daily morning peak commuting demands and the proportions of high, medium, and low-VOT groups for each year from 2025 to 2030. Different colors represent the share of each VOT group in the commuting population. (E) Average monthly fiscal revenue and carbon emissions from 2025 to 2030. The values shown in the figure are averaged over a six-month period.

The central urban area of Beijing, which houses 50.2% of Beijing’s permanent residents (Figure 6B) and is the major source of carbon emissions, is selected as the study region for the policy simulation. The morning peak travel system is chosen as the study’s focus. The Beijing government is assumed to implement the CCR policy from 2025 to 2030, with pricing aligned to the experimental setup. Assuming that individuals' initial mode choices in January 2025 align with the first cycle of the experiment and that their learning proceeds at the same rate observed in the experiment until convergence to a steady state, the evolution of the driving proportions is simulated for three groups. The criteria for determining whether a steady state has been reached are detailed in Section Policy simulation assumptions and methods. The simulation results indicate that the time required for each group to reach the stable condition varies (Figure 6A).

Income distribution and travel demand are critical determinants in forecasting carbon emissions and fiscal revenues. Data on the annual per capita disposable income of urban residents (five percentiles of the income distribution curve) and daily commuting demands on weekday mornings are collected from the Beijing Statistical Yearbook (2004–2022)^26^ and the Beijing Annual Traffic Report (2022–2023)^27^ to estimate these factors. Using the economic data, an ARIMA model is applied to forecast income percentiles, and income distribution curves are constructed for 2025–2030 under the assumption of log-normality (results shown in Figure 6C). The daily travel demand during morning peak hours is projected using linear interpolation (Figure 6D). Estimated results show that, from 2025 to 2030, the annual per capital disposable income will increase from 89,906 CNY to 100,560 CNY, while the daily commuting demand will increase from 6.79 million to 7.97 million trips. Then, the proportions of the low-, medium-, and high-VOT groups are derived based on the income distribution and travel demand.^10^ As shown in Figure 6A, these proportions remain stable over time.

Given the number of people in the three groups and their travel choices, Beijing’s monthly carbon emissions and revenues from 2025 to 2030 can be calculated, as shown in Figure 6E. Over the six-year period, total annual carbon emissions under the *Base*, *Int.1*, *Int.2*, and *Int.3* scenarios are projected to be 216,745, 220,866, 223,300, and 229,129 tons, respectively. Corresponding annual fiscal revenues are estimated at 0.538, 0.691, 0.822, and 1.037 billion CNY, respectively. Cost savings are defined as the additional fiscal revenue generated by combined interventions minus the capture cost of increased carbon emissions to comprehensively evaluate the effectiveness of interventions. Given that the cost of carbon capture ranges from several hundred to 1,000 USD per ton,^28^ this study adopts the upper bound of 1,000 USD per ton as a conservative estimate. Results indicate that over six years and considering only the morning peak, the cost savings for Beijing amount to 0.74 billion CNY, 1.42 billion CNY, and 2.47 billion CNY under *Int.1*, *Int.2*, and *Int.3*, respectively, compared to *Base*.

## Discussion

The findings highlight two key insights. First, under the CCR scheme, traveler behavior deviates from the theoretical predictions of full/economic rationality, particularly among high VOT participants. The logit model of high-VOT groups (Section Decision modeling) reveals that carbon credit levels exert a significant nonlinear influence on travel choices, reflecting participants' complex psychological calculus rather than pure economic rationality. Specifically, participants in a state of mild deficit ([-5, 0)) exhibit notable loss aversion, showing a strong inclination to choose public transit to avoid penalties. Conversely, those in a state of mild surplus ([0, 5]) demonstrate an increased probability of choosing to drive, with the magnitude of this increase exceeding the decrease observed under mild deficit conditions. Moreover, the probability of people choosing to drive increased with each round. These indicate that high-VOT individuals possess a strong preference for driving and have developed a sophisticated understanding of policy rules to strategically optimize their choices.

Second, the study demonstrates the substantial potential of non-fiscal interventions to enhance the CCR system’s fiscal sustainability and behavioral stability. While all three interventions improve the fiscal performance of the system, the downward comparison strategy (*Int.3*) exhibits the strongest role of behavioral regulation, particularly among high-VOT participants. According to the decision model (Section Decision modeling), the coefficient of C_[-5,0)_is smallest under *Int.3*, which may explain why *Int.3* has the strongest influence on high-VOT participants. This finding aligns with previous research on social comparison mechanisms that adjust people’s behavior,^29^ where downward comparisons increase rational behavioral adjustments. Compared to other strategies, participants under *Int. 3* do not exhibit habit persistence behavior with credit balances in [-20,-15). This can be attributed to the negative comparative feedback (i.e., having carbon credits lower than the quartile level) received by individuals within this credit range under *Int.3*, which highlights the drawbacks of *Int.3*. Notably, *Int.1* and *Int.3* show significant group-specific effects, whereas *Int. 2* has a stronger short-term outcome. This is consistent with prior findings that the effectiveness of nudges varies based on context and audience characteristics and that nudges' long-term roles differ from their short-term roles.^30^^,^^31^ These differentiated impacts highlight the importance of matching intervention types to specific population segments' behavioral patterns.

From a policy perspective, these findings have several important implications. Policymakers designing carbon reduction schemes must anticipate that cognitive biases, such as credit feedback overemphasis, may lead to behaviors that diverge from rational-choice predictions. Incorporating non-fiscal behavioral interventions—especially those leveraging social comparison mechanisms—helps correct such deviations and improves the system’s long-term sustainability and effectiveness. However, the differentiated impacts of the interventions across groups and time frames suggest that a one-size-fits-all approach is unlikely to be optimal. Instead, tailored combinations of strategies should be deployed to match the needs of diverse groups, and dynamic adjustments should be made over time. Moreover, care should be taken to monitor unintended psychological effects. For instance, when applying downward comparison strategies, it is essential to consider not only individual psychological effects—such as increased anxiety or demotivation among high-VOT drivers—but also broader societal implications. These may include the reinforcement of social inequalities or the stigmatization of certain groups, which could arise from repeatedly framing low-carbon-credit users as underperformers. To mitigate such risks, intervention strategies should prioritize personalization and inclusivity. This could involve, for example, providing comparative feedback only to users above the lower quartile of carbon credits, while designing supportive and empowering communication for those at the bottom—such as acknowledging their current low transportation costs and encouraging sustained effort. Moreover, policymakers should establish mechanisms for regularly monitoring the psychosocial impacts of interventions, particularly their long-term effects on environmental attitudes, social equity, and collective responsibility. An adaptive governance framework that integrates feedback and allows for iterative policy adjustments will be crucial to balance economic benefits with ethical considerations and avoid exacerbating structural disparities.

### Limitations of the study

The findings above should be considered while remaining cognizant of the study’s limitations. First, the online experiment format allows individuals to forecast outcomes that are completely different from what they actually choose in reality. Therefore, while these findings provide an indication of the CCR and nudge efficacy, this should be investigated in a real-life experiment or setting. Second, the study participants are primarily university students. While previous research demonstrates that university students' behaviors are qualitatively similar to those of the general population,^32^ there may be quantitative differences between this group and broader demographics. Such differences are associated with the accuracy of the simulation models. Therefore, field experiments should be conducted using more diverse and representative populations. Third, despite efforts to mitigate social desirability bias through some experimental designs, participants may infer that the experimenters favor environmentally friendly behavior due to the overtly environmental nature of the CCR policy and adjust their choices accordingly. Future experiments should improve the design by employing a double-blind or multiple-scenario task design.

In summary, this study advances the understanding of how individuals behave under a CCR framework and how non-fiscal behavioral interventions shape outcomes. By integrating behavioral science insights (particularly regarding bounded rationality, expert effect, and social comparison) into the design of carbon reduction policies, governments can more effectively tailor interventions to diverse populations and policy goals. Future research should expand these findings by conducting large-scale, real-world field experiments, exploring additional non-financial interventions such as boosts or pledges,^33^ and investigating the long-term roles of combining fiscal and non-fiscal strategies on both environmental outcomes and public acceptance.

While this study provides interventions to help the government control behavioral deviations, setting reasonable emission reduction and fiscal goals remains a prerequisite for the successful implementation of carbon credit policies. Therefore, policymakers must first establish emission reduction goals that balance environmental benefits with fiscal sustainability before implementing the CCR scheme.

## Resource availability

### Lead contact

Further information and requests for resources should be directed to and will be fulfilled by the lead contact, Zheng Zhu (zhuzheng89@zju.edu.cn).

### Materials availability

This study does not generate new unique materials.

### Data and code availability

The experimental data, the source code of the experimental platform, the scripts for four experiments (used for uploading to the platform to implement specific experiments), and the code to process the data, as well as train models, have all been publicly available at Zenodo (Zenodo: https://doi.org/10.5281/zenodo.17489256). The income data for Beijing is publicly available on the official website of the Beijing Municipal Bureau of Statistics.^26^ The transportation demand data for Beijing is publicly available on the official website of the Beijing Transport Institute.^27^ Any additional information required to reanalyze the data reported in this article is available from the lead contact upon request.

## Acknowledgments

The work described in this article was partially supported by the 10.13039/100014718Natural Science Foundation of China (72401255, 72525009, and 72431009), the 10.13039/501100011066Hong Kong Research Grants Council (HKUST16205123), the 10.13039/501100004731Natural Science Foundation of Zhejiang province, China (LZ25E080007), and the Smart Urban Future (SURF) Laboratory, Zhejiang Province.

## Author contributions

Conceptualization, C. H., Z. Z., H. D., H. Y., J. Z., Z. W., and G.H.; methodology, C. H., Z. Z., and J. Z.; platform development, testing, and optimization: Z. Z., C. H., and L. W.; experiments: C. H., L. W., Z. Z., and J. Z.; writing-original draft: C. H., K. W., L. W., and Z. Z.; writing-review and editing: C. H., Z. Z., K. W., and J. Z.; visualization, C. H., K. W., and Z. Z.; funding acquisition, Z. Z.

## Declaration of interests

The authors declare no competing interests.

## Declaration of generative AI and AI-assisted technologies in the writing process

During the preparation of this work, the authors used GPT4 in order to improve language and readability. After using this service, the authors reviewed and edited the content as needed and take full responsibility for the content of the publication.

## STAR★Methods

### Key resources table

**Table:** 

REAGENT or RESOURCE	SOURCE	IDENTIFIER
**Deposited data**		

Ecnomic data of Beijing (2004–2023)	Beijing Statistical Yearbook	Beijing Statistical Yearbook: https://tjj.beijing.gov.cn/EnglishSite/
Traffic demand data of Beijing(2022–2023)	Beijing Transportation Development Annual Report	Beijing Transportation Development Annual Report: https://www.bjtrc.org.cn/List/index/cid/7.html

**Software and algorithms**		

Python	Python Software Foundation	Python: https://www.python.org
All code used in this paper	This paper	Zenodo: https://doi.org/10.5281/zenodo.17489256

### Experimental model and study participant details

A total of 270 undergraduate and graduate students (51% male; 56% are undergraduate students;mean age = 23 years; self-reported ethnicity: 100% Asian) from Zhejiang University are recruited to participate in this experiment. All participants are randomly assigned to different experimental groups to ensure group equivalence and to minimize selection bias. The recruitment criteria are as follows: (1) participants must be at least 18 years old; (2) participants must possess normal vision or corrected-to-normal vision; (3) participants must be in good physical health with no major illnesses or interference from medications; (4) participants must have no history of mental or neurological disorders; and (5) participants must be able to understand and comply with the requirements of the study. The experimental procedures were approved by the Research Ethics Committee of College of Biomedical Engineering & Instrument Science, Zhejiang University (Reference Number: Zhejiang University Biomedical Engineering Ethics Review [2024] No. 5). All participants provided written informed consent before the experiment, and the possible consequences of the studies were explained.

### Method details

The major notations used afterward are listed in the following Table 2.

**Table 2:** List of major notations

Parameters	
*κ*_*a*_	Credit charge rate for each car trip
*κ*_*b*_	Credit reward rate for each transit trip
*p*_*c*_	Charging price per credit
*p*_*r*_	Redemption price per credit
*N*	Daily commuting demand
*t*_*a*_(*v*_*a*_)	Travel time function on the highway
*v*_*a*_	Daily system-wide highway flow
*τ*_*a*_	Monetary cost of each car trip
t_*b*_	Travel time of each transit trip
*τ*_*b*_	Monetary cost of each transit trip
*n*	A label for each traveler, numbered in a decreasing order of travelers' VOTs
*n*_*c*_	Traveler who is indifferent between car only and mixed mode
*n*_*r*_	Traveler who is indifferent between transit only and mixed mode
*β*(*n*)	VOT of the nth traveler
*θ*(*n*)	The driving frequency of traveler *n* in the period
*φ*_*c*_(*n*)	Traveler *n*’s credit deficit at the end of each period
*φ*_*r*_(*n*)	Traveler *n*’s credit surplus at the end of each period
$\bar{t_{a}\left(v_{a}\right)}$	Traveler's average driving time of each period
*g*(*n*)	Traveler *n*’s periodic travel cost, including time, fee, and credit costs
$\tilde{v_{a}}$	Daily highway flow at equilibrium without CCR schemes
$\bar{v_{a}}$	Daily highway flow at equilibrium with CCR schemes
*v*_*i*_	the average daily number of drivers in system during cycle/month *i*
*N*_h_, *N*_*m*_, *N*_*l*_	the number of high, medium and low VOT people, respectively
*P*_hi_, *P*_mi_, *P*_li_	the driving proportion of high, medium and low VOT people during month *i*
*l*_*a*_	the average commute distance
*E*_*i*_	CO2 emissions measured in *g*

*t*_*a*_(*v*_*a*_) is assumed to be continuous, convex, and monotonically increasing with *v*_*a*_.

#### Design of the experiment

A laboratory experiment is chosen to investigate behavioral responses under the CCR scheme to enable rapid deployment and precise variable control.

##### Theoretical equilibrium

The CCR scheme is implemented in a system with a fixed population of *N* people. The CCR scheme is announced to the public, including the credit charging and rewarding rates (κ_a_,κ_b_) and the credit charging and redemption prices (*p*_*c*_,*p*_*r*_). The travel time and monetary cost of each transit trip are denoted ast_*b*_ and*τ*_*b*_, while the travel time and monetary cost of each car trip are denoted as *t*_*a*_(*v*_*a*_) and*τ*_*a*_. There is a dual-mode commuting scenario, which is under a CCR strategy with parameters presented in Section Introduction. During the experimental phase, travelers' VOTs are categorized into three levels, whereas in the theoretical phase, a distribution is used to represent heterogeneity. All travelers are labeled by their VOTs in decreasing order, and *β*(*n*) denotes the VOT of the *n*th traveler. $\beta \left(n\right)=55-\frac{8}{3}n,n\in \left\{1,2,\ldots ,15\right\}\;\left(\text{CNY}/h\right)$ in this study. *θ*(*n*)∈[0,1] denotes the driving frequency of traveler n in the period. *φ*_*c*_(*n*) and *φ*_*r*_(*n*) represents traveler n’s credit deficit and surplus at the end of the period, respectively. $\bar{t_{a}\left(v_{a}\right)}$ denotes the traveler’s average driving time in the period. Equation 1 implies his travel cost over the period.

(1) $$g\left(n\right)=\theta \left(n\right)*20*\left(\beta \left(n\right)\bar{t_{a}\left(v_{a}\right)}+14\right)+\left(1\;-\;\theta \left(n\right)\right)\;*20*\left(\beta \left(n\right)*1+4\right)+3*\varphi_{c}\left(n\right)-1.5*\varphi_{r}\left(n\right)$$

Periodic mode usage equilibrium is defined as follows. At periodic mode usage equilibrium, the total travel costs of a cycle are minimized for each traveler, and no traveler can further reduce individual travel costs by unilaterally changing individual periodic mode usage patterns. Ding’s study^10^ has indicated that the system converges to different equilibrium with and without the CCR scheme.

Several conclusions, which are shown in Figures 3A and 3B, can be drawn according to a series of mathematical derivations^10^:

##### Derivation 1

Without a CCR scheme, the equilibrium car flow is $\tilde{v_{a}}=12.4$ 4 (vph). Travelers with VOT = *β*(*n*) > 22 choose to drive for each trip and those with VOT = *β*(*n*) < 22 choose to transit for each trip. These two groups account for 82.5% and 17.5% of the population, respectively. The average per capital carbon emission in the system is 77.1 g.

##### Derivation 2

Under the CCR scheme, the critical VOTs are (*β*(*n*_*r*_),*β*(*n*_*c*_)) = (28.3, 41.6), where *n*_*c*_ represents the traveler who is indifferent between car only and mixed mode. Meanwhile, *n*_*r*_ represents the traveler who is indifferent between transit only and mixed mode. Travelers with *β*(*n*) between 29.8 and 42.5 are mixed-mode travelers (accounted for 1/3); their driving frequency is constant and equal to *κ*_*b*_/(*κ*_*a*_+*κ*_*b*_) = 0.6, in other words, 60% of the daily mixed-mode commuters are drivers. Travelers with VOT = *β*(*n*) > 42.5 (accounted for 1/3) choose to drive for each trip and those with VOT = *β*(*n*) < 29.8 (accounted for 1/3) choose to transit for each trip. These three groups are referred to as low, medium, and high VOT groups, respectively. Overall, 53.3% of people are drivers, which demonstrates that the CCR effectively promotes the use of public transportation. The government can get a revenue of 10 CNY per person per cycle and have a carbon emission level of 31.5 g per person per cycle. Derivation 2 presents the predicted results of the experiments.

For simplicity, the travelers' VOT values in the experiments are set at three representative levels: 20, 36, and 50 CNY per hour, called the high-, medium-, and low- VOT group, respectively. Therefore, the participants of each experiment are evenly divided into three groups. The reseachers have informed participants of their time value through the platform interface. Notably, in order to enhance participants' sense of immersion, a survey is conducted on their monthly living expenses before the experiments as the reference to divide groups. However, due to the uneven distribution of living expenses among the recruited participants, 30% are assigned to groups that did not align with their actual time value.

##### Base

The reported highway travel time influences the subjective forecast of future driving time. The travel costs and the remaining tokens are two representations of the travel costs and serve as key information for fully rational participants. For instance, if choosing the highway on a certain day leads to higher costs, a fully rational participant will switch to public transportation the next day to maximize their overall score. Participants update their decisions day by day based on travel costs or remaining tokens and cycle by cycle based on the average score from previous rounds. In contrast, the carbon credit balance is a less critical piece of information for fully rational participants. It is associated with but does not have a direct influence on travel costs. Indeed, a lower remaining carbon credit balance may even lead to a higher final score for participants with a high VOT.

##### Expert recommendation (Int. 1)

Expert recommendations, such as adopting energy-efficient appliances and sustainable diets, have been shown to effectively influence environmental decisions.^34^^,^^35^ The theoretical underpinnings of their effectiveness draw on cognitive load theory,^36^ cognitive inertia,^37^ and the authority effect.^38^ These recommendations reduce the cognitive burden associated with complex decision-making, counteract the tendency of individuals to rely on prior habits, and enhance compliance by leveraging perceived expertise.

As shown in Figure 2C (*Int*.1), a new metric “expert-recommended mode today” is added to the daily feedback. Additionally, participants are informed during the introduction of *Int*.1 that following the expert-recommended mode results in minimal travel costs. The recommended modes (Table S2) align with the theoretical predictions.

##### Top-score feedback (Int. 2)

Rank-based incentives are originally conceptualized in educational and economic contexts, leveraging the mechanisms of social comparison.^39^ They are applied to other fields such as energy conservation and health. Social comparisons can be categorized into two types: upward and downward comparisons.^24^ Upward comparisons, such as corporate ranking systems designed to incentivize employee effort, typically serve to enhance motivation by encouraging individuals to emulate higher-performing peers.^40^ Downward comparisons are often used to alleviate psychological stress, where individuals derive reassurance from the perception that their situation is better than others, such as reducing comorbid anxiety in patients.^41^

In recent years, upward comparisons have been increasingly applied in environmental research to promote eco-friendly behavior. In this study, the participants are motivated to align their behavior with that of high-score individuals through upward comparisons. As illustrated in Figure 2C (*Int.2*), a new metric, “highest value of remaining tokens in historical data,” is added to the daily feedback information. The reference metric is initially derived from the *data of* the *Base session* and then iteratively updated each cycle based on the current participants' performance.

##### Low-credit feedback (Int. 3)

The results from the *Base* experiment indicate the high-VOT group as the primary source of bounded-rational behaviors. It is speculated that the phenomenon stems from negative emotions triggered by perceived CCLs associated with carbon credit losses associated with the choice of driving. Therefore, *Int*.3 only targets the high-VOT group, which displays the third quartile of credits, guiding participants to make downward comparisons and enhancing their sense of self-confidence. As shown in Figure 2C (*Int.3*), a metric, “the lower quartile value of remaining carbon credits in historical data”, is added to the daily feedback information. The reference value is initially derived from the *Base* experiment data and then iteratively updated each cycle based on the current performance of the participants.

#### Implementation of the experiment

##### Platform

An interactive experimental platform is developed that allows multiple participants to engage in collaborative and day-to-day decision-making online (one mouse click represents the decision of one day). Experimenters create new experiments by writing Python scripts, and participants make choices through mouse clicks with reference information (parameters, pictures, and so forth) shown to them. After all participants have made mouse clicks on a day, the system (python scripts) calculates the feedback parameters based on both the collective choices and the individual’s own decisions, resulting in personalized feedback for each participant. For instance, if 6 participants choose drive and 9 choose transit, then *v*_*a*_ = 6 and *t*_*a*_(*v*_*a*_) = 23 min; and a high-VOT participant who drives with a credit of 1 at the beginning of the day will get a 23 min travel time, a 14 CNY fee, a 2 credits reduction, and a 37.7 CNY total cost on this day. The cost due to changes in carbon credits equals the difference between the cost generated by settling with the reduced carbon credits and the cost generated by settling with the original carbon credits. The participants proceed to choose the next day mode choice based on the feedback parameters. At the end of each cycle, the reseachers have informed the participants about their scores. The system automatically records each participant’s choices and reference parameters every day. (Figures S3 and S4).

##### Procedures for the experiment

The experiments are conducted in a computer laboratory with multiple terminals. Adequate spacing is ensured to prevent discussion among participants. Before the formal experiment, a 20-min slide presentation introduces the CCR rules, feedback information, the tasks, and the components of the payout. Each participant then signs an informed consent form Questions about the procedures are answered individually for each participant. Then, the experiments begin. Each experiment lasts approximately 60 min.

At the end of each cycle, participants are provided with a certain number of tokens specific to their VOT groups. Scores for each cycle are calculated by subtracting travel costs from the initial tokens. Cumulative scores across cycles are converted to CNY at a fixed ratio. This payout, along with a 25 CNY show-up fee, constitutes each participant’s total reward (Table S1).

The researchers have informed the participants that the CCR policy background is related to emission reduction, which may lead them to speculate that the purpose of the experiment is to test pro-environmental behavior, thereby increasing their use of public transportation. To mitigate this bias, control measures are implemented through the incentive structure: it is emphasized that participants' goals are to maximize their own scores, and the score-based reward accounts for two-thirds of the total compensation when introducing the experiments, encouraging a focus on cost minimization over social conformity. This effect can be offset in between-group comparative analyses.

#### Logit model

The formula of the logit model in Section decision modeling (questions 1 and 2) is as follows:

(2) $$\text{logit}\left(P\right)=\log\left(\frac{P}{1-P}\right)=\beta_{0}+\beta_{1}{\text{Cost}}_{1}+\beta_{2}{\text{Cost}}_{2}+\beta_{3}C_{\left[-5,0\right)}+\beta_{4}C_{\left[-20,-15\right)}+\beta_{5}C_{\left[0,5\right)}$$

where *P* represents the probability of choosing to drive, *β*_0_ denotes the constant term, and *β*_1_–*β*_5_ represent the coefficients of each explanatory variable. *Cost*_*1*_ and *Cost*_*2*_ are numerical variables denoting the cost of driving and subway transit, respectively.*C*_[-5,0)_,*C*_[-20,-15)_,*C*_[0,5)_ represent three credit-related dummy variables (0/1). Parameter estimates are obtained using the maximum likelihood method. A positive coefficient shows a positive relationship between the variable and car usage, vice versa.

#### Policy simulation assumptions and methods

The steps in the simulation include predicting the driving proportions, forecasting income distributions, forecasting travel demand, calculating the population of the three groups, and calculating carbon emissions and fiscal revenues.

Given that the methodologies in other parts are relatively straightforward, only the methods used for predicting the driving proportions and calculating the population of the three groups are clarified. The income prediction results are provided in Tables S5 and S6. Sensitivity analysis on key contextual parameters, including household income levels and travel demands, is conducted, showing that the absolute value of income has little impact on cost savings estimates, while travel demand exhibits a positive correlation with cost savings (Figures S1 and S2).

Assumptions the simulation relies on several fixed assumptions that inevitably introduce a certain degree of speculation. Specifically, the assumptions uses in this study (along with their respective limitations and rationales) are summarized in Table 3. These assumptions are grounded in solid theoretical and empirical foundations derived from previous research and are based on conservative estimates. This approach provides a reliable reference for assessing the magnitude of effects and enables robust comparative analysis. For instance, an ARIMA model is employed to extrapolate residents' income over the next five years, suggesting a largely unchanged income structure; and this is align with numerous studies, claiming that China’s income structure is expected to undergo continuous yet moderate adjustments rather than drastic changes in the next five years. Despite potential inaccuracy induced by behavioral scaling from experimental to real-world contexts, these assumptions can limit the prediction error into an acceptable range. And a modeling analysis is added in the supplementary materials to discuss how factors such as age, income, and gender affect people’s choices under the CCR policy (Table S3).

**Table 3:** Summary of assumptions in the case of Beijing

Category	Specific assumption	Basis and rationality	Associated uncertainties
Behavioral Choice	Laboratory-based behavioral learning curves and convergence rates can be directly extrapolated to the real world.	The experimental environment controls core variables, providing an “ideal” baseline rate for behavioral change.^42^	Factors such as inertia, fatigue effects, social pressure, and information friction may slow the convergence rate in real-world contexts.
	The behavioral responses and patterns observed in the student participant sample are representative of and generalizable to the broader population.	Students are readily available and have the intellectual capacity to understand complex instructions.^43^	Students may differ from the general population in terms of age, income, risk preferences, cognitive ability, and flexibility to adopt new behaviors.
	When the rate of change falls below a certain threshold, the behavioral change has reached its limit.	Accounts for saturation in behavioral change (early adopters change quickly, while the remaining individuals may be “hard-to-reach” groups with the lowest willingness to change^42^).	Real-world situations must consider structural constraints (e.g., individuals who must rely on cars for work).
Policy Settings	Policy pricing is consistent with the experimental setup.	Enhances the extrapolation plausibility.^44^	Actual policy pricing involves complex political and economic evaluations.
Income Structure	The income distribution follows a log-normal distribution.	A commonly used economic assumption.^45^	Despite reasonability, model fitting always involves errors.
	The income structure is relatively stable.	Historical data projections exhibit a certain degree of stability.^46^	Economic shocks or urban policies may alter income distribution.
Transportation Context	Commuting patterns are assumed to remain largely unchanged over the next five years.	Serves as a baseline simulation for a bimodal scenario.	The real-world commuting patterns may be more diverse.^47^
	Commuting demand grows linearly	Based on simple projections of historical trends.	Urban development (on transit infrastructures) may exhibit nonlinear growth.

##### Prediction of driving proportions

As the driving proportions for each VOT group exhibit a converging trend toward a predicted value, an exponential curve is employed for fitting and prediction:

(3) $$F_{low}\left(x;a,b\right)=a_{low}\times \exp\left(-b_{low}x\right)$$

(4) $$F_{med}\left(x;a,b\right)=0.6-a_{med}\times \exp\left(-b_{med}x\right)$$

(5) $$F_{high}\left(x;a,b\right)=1-a_{high}\times \exp\left(-b_{high}x\right)$$

Equations 2, 3, and 4 serve as the respective fitting functions for the low-, medium-, and high-VOT groups, where the parameters to be estimated are denoted. The fitting process follows the principle of least squares (see Table S4 for fitting outcomes).

Given that driving proportions do not asymptotically reach theoretical values, a threshold value is defined: a system is considered stable if the month-to-month change or the deviation from the theoretical prediction in driving proportions falls below a defined threshold. It is assumed that groups or strategies with poor performance at the end of the experiments likewise converge to suboptimal values in real-world settings. Accordingly, the threshold is defined based on the deviation between the stable driving frequency and the corresponding theoretical value, varying by group and strategy (more details in Table S5). The final results are presented in Figure 6A.

#### Calculation of the population for the three groups

(6) $$\beta \left(n_{c}\right)=\frac{p_{c}\left(\kappa_{a}+\kappa_{b}\right)+\left(\tau_{a}-\tau_{b}\right)}{t_{b}-t_{a}\left(\bar{v_{a}}\right)}$$

(7) $$\beta \left(n_{r}\right)=\frac{p_{r}\left(\kappa_{a}+\kappa_{b}\right)+\left(\tau_{a}-\tau_{b}\right)\;}{t_{b}-t_{a}\left(\bar{v_{a}}\right)}$$

(8) $$\bar{v_{a}}=\frac{\kappa_{a}n_{c}+\kappa_{b}n_{r}}{\kappa_{a}+\kappa_{b}},0\leq n_{c}\leq n_{r}\leq N$$

(9) $$N_{h}=n_{c}$$

(10) $$N_{m}=n_{r}-n_{c}$$

(11) $$N_{l}=N-n_{r}$$

Where $\bar{v_{a}}$ denotes the daily highway flow at equilibrium with CCR schemes. The income-based method assumes that the VOT correlates positively with income levels. The VOT distribution mirrors the income distribution according to the income approach, which is widely used to evaluate the VOT. *N*_*h*_, *N*_*m*_, and *N*_*l*_ present the number of high, medium and low VOT people, respectively. A person typically makes only one morning commute per day, so *N* equals the number of daily morning commuting demands. With the known CCR pricing and the system’s VOT distribution, Equations 4, 5, 6, 7, 8, and 9 form^10^ a deterministic system of equations with a unique solution for the *N*_*h*_, *N*_*m*_, and *N*_*l*_. The calculation results for 2025–2030 are presented in Figure 6C Based on the driving proportions and the population of different groups, Beijing’s daily number of drivers in the system during month *i* is able to be calculated:

(12) $$v_{i}=N_{hi}\times P_{hi}+N_{mi}\times P_{mi}+N_{li}\times P_{li}$$

where *N*_*hi*_, *N*_*mi*_, *N*_*li*_ present the number of high, medium and low VOT people during month *i, P*_*hi*_*, P*_*mi*_*, P*_*li*_ present the driving proportion of high, medium and low VOT people during month *i, v*_*i*_ is the average daily number of drivers in the system during cycle/month *i*, which is the core variable for calculating carbon emissions and fiscal revenue.

#### Carbon emission calculation

For the calculation of carbon emissions, the emission function proposed by Yin and Lawphongpanich^48^ is adopted in this study, expressed as:

(13) e(vi)=0.2038×ta(vi)×e0.7962×lata(vi)

Where *l*_*a*_ presents the average commuting distance of the system.*l*_*a*_ = 11.6 km in the case.^27^ Results are measured in g/h and represented CO emissions. Previous researches indicate that CO2 emissions from vehicle exhaust in China are typically 10 to 100 times higher than CO emissions.^49^^,^^50^^,^^51^ A conservative value of 80 is adopted. Then, the carbon emission for cycle *i* is:

(14) $$E_{i}=e\left(v_{i}\right)\times v_{i}\times t_{a}\times 20\times 100/N$$

where *E*_*i*_ presents per capital CO2 emissions measured in *g*.

### Quantification and statistical analysis

Statistical analysis is performed using Python software. *t* test hypothesis testing is utilized to evaluate the stable metrics. The Mann-Whitney U test is applied to assess dynamic metrics. A *p*-value of less than 0.05 is deemed significant.The significance levels are represented as “ns” for *p* > 0.05. In the boxplot (Figure 4), the center line represents the median, the box spans the interquartile range (IQR) from the 25th to the 75th percentile, and the whiskers extend to the minimum and maximum values within the dataset.
