# Supplementary material for: Downward comparisons enhance carbon credit policy sustainability: Lab evidence
Source: iScience. 2025 Nov 13;28(12):114050. doi: 10.1016/j.isci.2025.114050 (PMC12721203; doi:10.1016/j.isci.2025.114050)
Supplement: Document S1. Figures S1–S4 and Tables S1–S6 [file mmc1.pdf]

iScience, Volume *28*

Supplemental information

Downward comparisons enhance carbon credit policy sustainability: Lab evidence

Chenke Hu, Kehua Wang, Linghao Wang, Jun Zhao, Zheng Zhu, Hai Yang, Hongxing Ding, Guibing He, and Ziyi Wang

Table S1. values of X and α.

	VOT = 20 CNY/h	VOT = 36 CNY/h	VOT = 50 CNY/h
X (CNY)	480	800	980
α	100:9	100:6.5	100:5

Notes:At the conclusion of the experiment, the cumulative earnings from six cycles will be converted into cash at a rate of α. The value of X (tokens awarded per cycle) and the final conversion ratio α vary across different VOT groups, as presented in Supplementary Table 1. However, the average rewards for all groups are ensured to be approximately equal.

Table S2. The recommendation schemes in *Int.2.*

Day of cycle	Recommend 'car' to these participants	Day of cycle	Recommend 'car' to these participants
Day1	A, B, C	Day11	A, D, E
Day2	A, B, C	Day12	A, D, E
Day3	A, B, D	Day13	B, C, D
Day4	A, B, D	Day14	B, C, D
Day5	A, B, E	Day15	B, C, E
Day6	A, B, E	Day16	B, C, E
Day7	A, C, D	Day17	B, D, E
Day8	A, C, D	Day18	B, D, E
Day9	A, C, E	Day19	C, D, E
Day10	A, C, E	Day20	C, D, E

In *Int.2*, we consistently recommend 'transit' to the low-VOT group and 'car' to the high-VOT group. For the medium-VOT group, the five participants involved together are randomly assigned labels A to E, and the recommendation scheme is designed as shown in Supplementary Table 2. This scheme satisfies the following conditions: (1) 60% of participants choose to drive each day, and (2) each participant has a 60% probability of choosing to drive.

Table S3. Summary of logit model

All experiments	Sample = 13680, R^2=0.350				
	**Coef.**	**Std.Err.**	**[0.025**	**0.975]**	**p-value**
**Const.**	0.21	0.10	0.01	0.41	0.04
$\mathbf{high}\_C_{[-5,0)}$	0.43	0.09	0.25	0.62	0.00
$\mathbf{high}\_C_{[-20,-15)}$	0.14	0.11	-0.08	0.35	0.22
$\mathbf{high}\_C_{[0,5)}$	4.28	0.26	3.77	4.80	0.00
$\mathbf{low}\_C_{[-5,0)}$	1.61	0.29	1.04	2.18	0.00
$\mathbf{low}\_C_{[0,5)}$	0.08	0.12	-0.15	0.30	0.49
$\mathbf{med}\_C_{[-5,0)}$	1.50	0.08	1.35	1.66	0.00
$\mathbf{med}\_C_{[-20,-15)}$	0.30	0.06	0.17	0.43	0.00
$\mathbf{med}\_C_{[0,5)}$	3.22	0.24	2.75	3.69	0.00
**Cost**	-2.51	0.06	-2.64	-2.39	0.00
**Gender (male)**	-0.13	0.05	-0.22	-0.04	0.00
**Age**	-0.13	0.09	-0.31	0.06	0.78
**Income**	0.28	0.03	0.21	0.35	0.00

Notes:Std.Err.: The standard error of the coefficient estimate. [0.025 | 0.975]: The lower and upper bounds of the 95% confidence interval for the coefficient.The effects of gender and income are statistically significant, though the coefficient magnitudes are not large. When the individual is male, the proportion choosing to drive is smaller. This suggests that under the CCR policy, males may exhibit higher sensitivity to costs or carbon credit mechanisms. As monthly income increases, the probability of choosing to drive also increases. This confirms that economic capacity is a key factor influencing travel mode choice, and variations in income distribution may lead to observable shifts in commuting patterns at the aggregate level.

As this study is an online experiment, the demographic information that could be collected was limited. Objective environmental variables—such as household size, commuting distance, and public transport accessibility—were difficult to accurately obtain or simulate in an experimental setting. We acknowledge that omitting these built environment and household structure factors is a limitation. These variables likely shape final travel decisions by influencing individuals' feasible choice sets (e.g., people without a subway station nearby cannot choose metro) and travel demands (e.g., households with children rely more on private cars). Future research

should, where feasible, control for the influence of these factors by collecting richer individual-level data or integrating geographic information system (GIS) data, thereby providing a more refined estimation of the impact mechanisms of CCR policies.

Table S4. Fitting results and fit-goodness of the driving proportion curves, as well as the of different conditions.

Group	Strategy	a	b	Log-MSE	Threshold1	Threshold2
VOT=20	*Base*	0.079639	0.130398	0.000233	0.001	0.001
	Int.1	0.052494	0.096680	0.000078		
	Int.2	0.161015	0.444263	0.000018		
	Int.3	0.282000	0.632977	0.000089		
VOT=36	*Base*	0.069088	0.174494	0.0000099		
	Int.1	0.116439	0.308877	0.000163		
	Int.2	0.050257	0.263147	0.000038		
	Int.3	0.164402	0.510158	0.000063		
VOT=50	*Base*	0.267810	0.159484	0.000055	0.01	0.004
	Int.1	0.152137	0.084081	0.000036		
	Int.2	0.207936	0.141522	0.000178	0.004	0.004
	Int.3	0.155840	0.263226	0.000057		

*Threshold1 was set for the difference in driving proportions between consecutive months, while Threshold2 was set for the difference in driving proportions between the predicted value and the theoretical expectation

Table S5. Fitting results and fit-goodness of the ARIMA models for the income.

Income quantiles	model parameters F（p, d, q）	R^2
20%	(4,3,0)	0.9328
40%	(4,2,0)	0.9617

60%	(6,3,0)	0.9292
80%	(5,2,2)	0.9622
100%	(5,2,0)	0.9580

Table S6. Fitting results and fit-goodness of the log-normal functions for the incomes.

year	model parameters (μ,σ)	R^2
2025	(11.39, 0.593)	0.996
2026	(11.43, 0.595)	0.995
2027	(11.46, 0.600)	0.995
2028	(11.49, 0.606)	0.995
2029	(11.52, 0.612)	0.994
2030	(11.55, 0.617)	0.993

* The probability density function of a log-normal distribution is expressed as:

$$f(x;\mu,\sigma) = \frac{1}{x\sigma\sqrt{2\pi}}\exp(-\frac{(\ln(x)-\mu)^2}{2\sigma^2}), \qquad x > 0$$

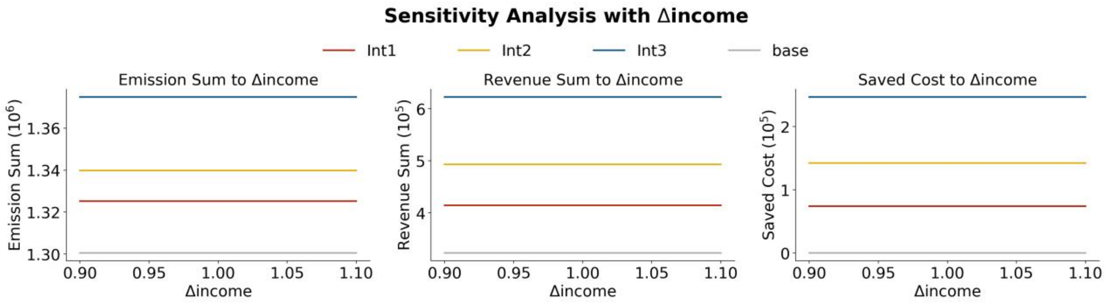

Figure S1. **The sensitivity analysis results of the average annual per capital disposable income.** Including the sensitivity analysis results of total carbon emissions with respect to the income, the sensitivity analysis results of total fiscal revenue to the income, the sensitivity analysis results of total cost savings to the income.

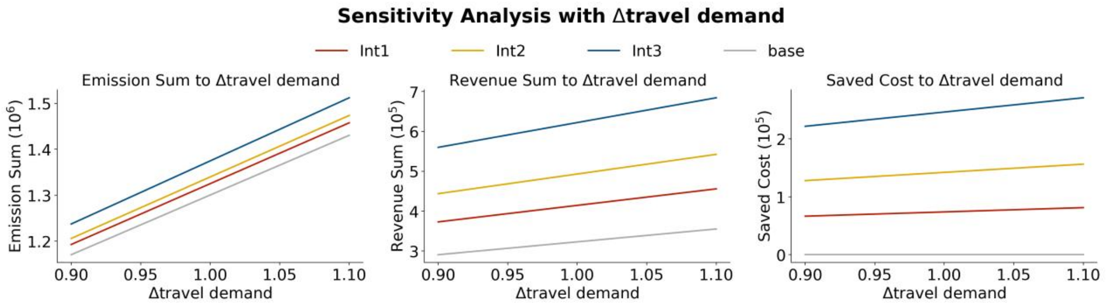

Figure S2. **The sensitivity analysis results of the average daily commuting demand.** Including the sensitivity analysis results of total carbon emissions to the demand, the sensitivity analysis results of total fiscal revenue to the demand, the sensitivity analysis results of total cost savings to the demand.

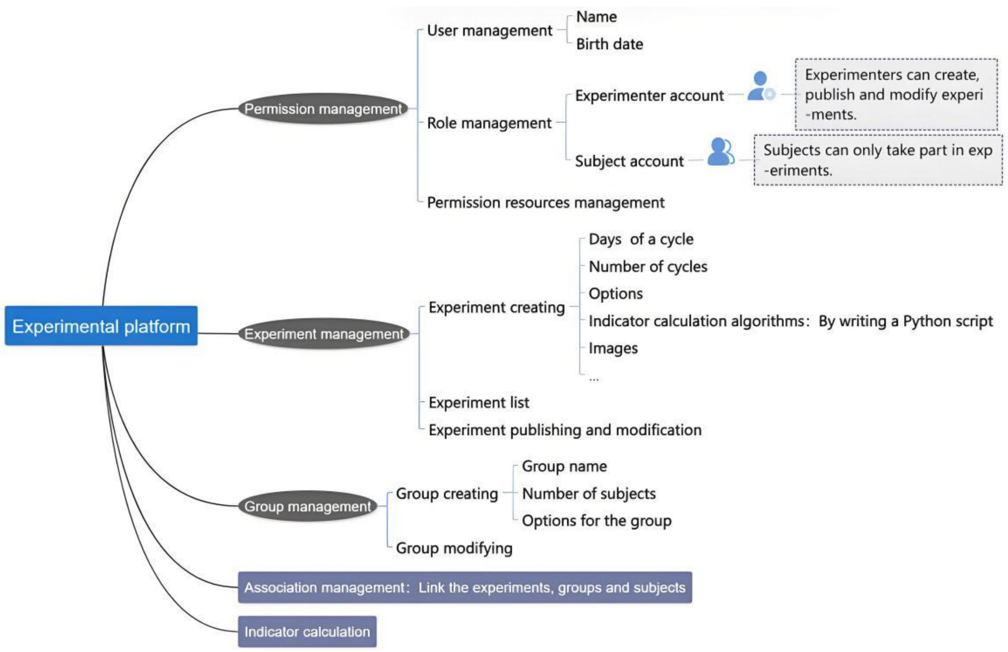

Figure S3. Functions of the experiment platform.

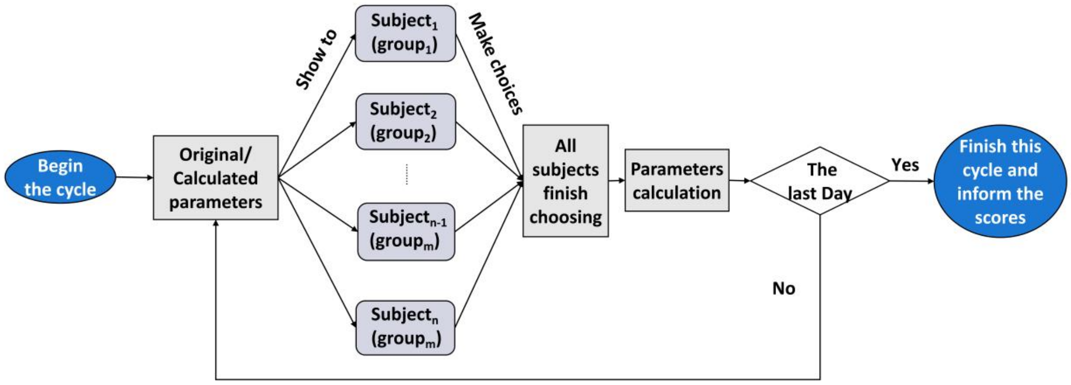

Figure S4. The interaction design of the experiment platform.
